# Drug Nanoparticle Formulation Using Ascorbic Acid Derivatives

**DOI:** 10.1155/2011/138929

**Published:** 2011-04-26

**Authors:** Kunikazu Moribe, Waree Limwikrant, Kenjirou Higashi, Keiji Yamamoto

**Affiliations:** Graduate School of Pharmaceutical Sciences, Chiba University, 1-8-1, Inohana, Chuo-ku, Chiba 260-8675, Japan

## Abstract

Drug nanoparticle formulation using ascorbic acid derivatives and its therapeutic uses have recently been introduced. Hydrophilic ascorbic acid derivatives such as ascorbyl glycoside have been used not only as antioxidants but also as food and pharmaceutical excipients. In addition to drug solubilization, drug nanoparticle formation was observed using ascorbyl glycoside. Hydrophobic ascorbic acid derivatives such as ascorbyl mono- and di-n-alkyl fatty acid derivatives are used either as drugs or carrier components. Ascorbyl n-alkyl fatty acid derivatives have been formulated as antioxidants or anticancer drugs for nanoparticle formulations such as micelles, microemulsions, and liposomes. ASC-P vesicles called aspasomes are submicron-sized particles that can encapsulate hydrophilic drugs. Several transdermal and injectable formulations of ascorbyl n-alkyl fatty acid derivatives were used, including ascorbyl palmitate.

## 1. Introduction

Antioxidants protect living systems against lipid peroxidation. Vitamin E (tocopherol) and vitamin C (ascorbic acid) are well-known lipophilic and hydrophilic chain-breaking antioxidants, respectively [1]. Because antioxidant activity in homogeneous solutions may not be the same as that in heterogeneous solutions, the antioxidant properties of heterogeneous solutions including aggregated systems (micelles, liposomes, and microemulsions) have been investigated. Variation of biomembrane microenvironments may turn vitamin E into a pro-oxidant agent [2]. 

**Figure 1:**
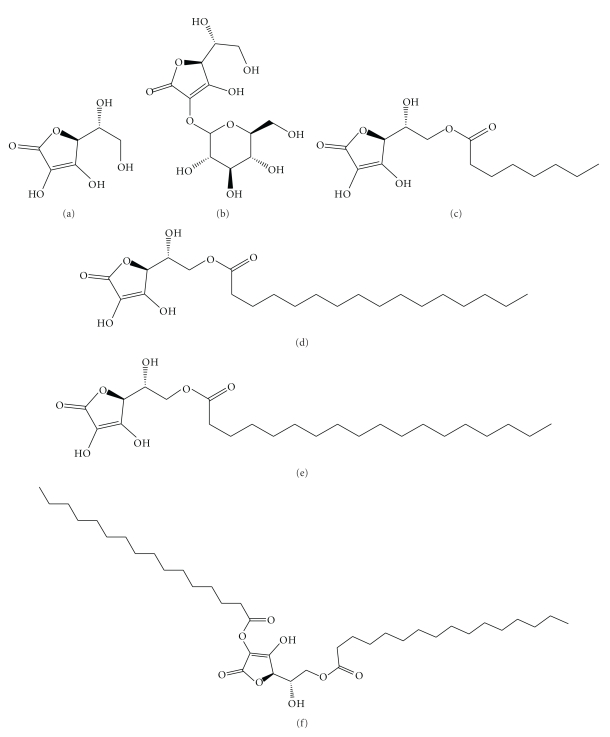
Chemical structures of ascorbic acid and its derivatives: (a) ascorbic acid (ASA), (b) ascorbyl-2-glucoside (ASC-G), (c) ascorbyl-6-octanoate (ASC-8), (d) ascorbyl-6-palmitate (ASC-P), (e) ascorbyl-6-stearate (ASC-S), and (f) ascorbyl-2,6-dipalmitate (ASC-DP).

Ascorbic acid contains hydroxyl groups in positions 2 (pK_a_ : 11.6), 3 (pK_a_ : 4.2), 5 (secondary alcoholic residue), and 6 (primary alcoholic residue) (Figure 1). Ascorbic acid is an ineffective antioxidant for lipid peroxidation in hydrophobic phases, but it works very efficiently in aqueous media [2]. Structural modification of position 2, 3, 5, or 6 of the ascorbic acid ring contributes not only to its stabilization as an antioxidant but also to the formulation of a variety of pharmaceutical and cosmetic products with antioxidant activity.

Ascorbic acid derivatives can retain the same activity exhibited by ascorbic acid. For example, the antioxidant activity of O-substituted ascorbic acid derivatives at the C-2 position—ascorbic acid 2-glucoside, ascorbic acid 2-phosphate, and ascorbic acid 2-sulfate—was investigated by Takebayashi et al. [3]. The radical-scavenging profiles of ascorbic acid derivatives were closer to those of uric acid and glutathione than to that of ascorbic acid. These data suggest the potential usage of ascorbic acid derivatives as radical scavengers. 

Hydrophobic ascorbic acid derivatives, in which one or more hydrocarbon chains are attached to the ascorbic acid ring, retain the activity displayed by ascorbic acid. The antioxidant activity of various 6-O-alkanoyl-ascorbic acids is much better than that of ascorbic acid and tocopherols both in vivo and in vitro [1]. The role of longer alkyl chains in facilitating the insertion of ascorbic acid derivatives into the cellular bilayer broadens its use to nonaqueous media. The addition of hydrocarbon chains (e.g., ethers and esters at positions 2, 3, 5, or 6 of the ascorbic acid ring) results in the formation of amphiphilic structures in which ascorbic acid can produce self-assembled supramolecular aggregates such as micelles and vesicles.

In this paper, drug nanoparticle formulation using ascorbic acid derivatives is introduced. Hydrophilic ascorbic acid derivatives have been used not only as antioxidants but also as food or pharmaceutical excipients [4, 5]. They are usually loaded into a nanoparticle formulation to prevent oxidation of the drugs and the components [4, 6]. Ascorbyl n-alkyl fatty acid derivatives have been well investigated as antioxidants for nanoparticle formulations, such as micelles, microemulsions, and liposomes. Physicochemical properties of ascorbic acid derivatives described in the paper and their applications are summarized in Tables 1 and 2, respectively. Ascorbyl-2-glucoside and ascorbyl palmitate have been well investigated among the derivatives. Physicochemical property, especially, solubility of ascorbyl acid derivatives was apparently changed not only by the substitution of alkyl chains but also by the chain length. Several papers have described ascorbic acid derivatives including the current methods of synthesis [7], so we hereby focused on the nanoparticle formulations themselves.

### 1.1. Ascorbyl-2-Glucoside (ASC-G)

**Figure 2:**
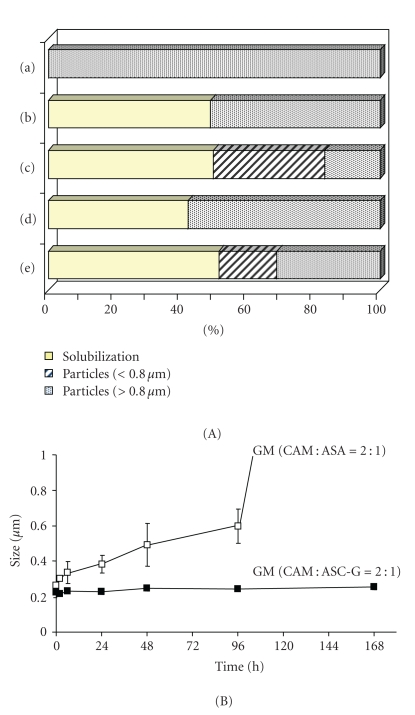
Comparison of solubilization and nanoparticle formation characteristics between the CAM-ascorbic acid (ASA) and CAM-ASC-G systems. (A) Particle fractions of the drug in the CAM : ASC-G systems. (B) Changes in the mean particle size of CAM fine particles after storage: (a) Unprocessed CAM, (b) PM (CAM : ASC-G = 2 : 1), (c) GM (CAM : ASC-G = 2 : 1), (d) PM (CAM : ASA = 2 : 1), and (e) GM (CAM : ASA = 2 : 1).

As a 2-O-substituted ascorbic acid, ASC-G was used as not only a solubilizer but also as an additive for nanoparticle formation. ASC-G has 2 beneficial properties: high stability against thermal and oxidative degradation and rapid conversion to ascorbic acid by *α*-glucosidase in blood and liver cells [8, 9]. ASC-G is a newly developed food additive. Moreover, it is expected to be used in the development of lipid-soluble vitamins and as the principal component in cosmetic ingredients [10]. Inoue et al. reported solubilization and nanoparticle formation of clarithromycin (CAM) using ASC-G [11]. We used ascorbic acid as a solubilizing agent because it can solubilize CAM; however, photodecomposition of ascorbic acid was observed in aqueous media. To avoid photodecomposition, ASC-G was used instead of ascorbic acid to improve the dissolution characteristics of CAM (Figure 2). Cogrinding of CAM with ASC-G at a molar ratio of 1 : 1 or less was an effective way to improve CAM solubility in aqueous solution. Molecular interaction between the N,N-dimethyl group of CAM and the hydroxyl group of ASC-G in aqueous solution was observed by ^1^H and ^13^C nuclear magnetic resonance (NMR). In addition to solubilization, CAM nanoparticle formation was observed when a 2 : 1 ground mixture of CAM and ASC-G was dispersed into an aqueous media. This molar ratio-specific nanoparticle formation might be attributable to a grinding-induced interaction in the solid state via the ketone group in the lactone ring of CAM. It is concluded that cogrinding with ASC-G is a promising method for modifying the dissolution properties of CAM. Further study focusing on the application of ASC-G in other poorly water-soluble drugs is required.

### 1.2. Ascorbyl n-Alkyl Fatty Acid Derivative: Structure and Oxidation Process

Ascorbyl monoalkylate has both lipophilic and hydrophilic moiety and exhibits properties of typical surfactant. The structures and physicochemical properties have been well described by Palma et al. [12]. The self-assembly properties depend on the length of the n-alkyl fatty chain. Ascorbyl monoalkylate starts to aggregate at the Krafft point, at which the solubility reaches the critical micellar concentration (CMC). Above this temperature, ascorbyl monoalkylate can aggregate in micelles or the gel phase, depending on the alkyl side chain. Upon cooling, liquid-crystal structures (coagels) are obtained for less soluble derivatives (ascorbyl laurate, ascorbyl myristate, and ascorbyl palmitate). These structures can solubilize drugs, improve their stability, and promote their permeation through the skin. Their rheological properties are also suitable for topical administration of pharmaceuticals.

**Figure 3:**
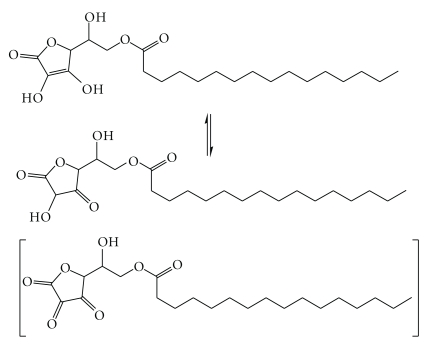
Tautomerization of ascorbyl palmitate. Structure of dehydroascorbyl palmitate is shown in parentheses.

Reducing activity (RA) measurement of some antioxidant chemicals indicated that hydrophobic vitamin C derivatives (from ascorbyl octanoate to ascorbyl stearate) keep the same antioxidant activity of vitamin C but have the advantage of being soluble in both aqueous and hydrophobic media [1]. These derivatives possess the same RA of several natural products; therefore, they can be used as radical scavengers in the protection of such natural compounds. The enediol functionality of ascorbyl palmitate is prone to oxidation in the presence of oxygen, a process that usually leads to formation of dehydroascorbyl palmitate (Figure 3) in which the OH functionality is oxidized into keto moiety [13–15].

### 1.3. Ascorbyl Octanoate (ASC-8)

The self-assembling and antioxidant activities of ASC-8 were reported by LoNostro et al. [1]. ASC-8 formed homogeneous micelles in pH 2 aqueous solution at 25°C (nonionic form), exhibiting nearly spherical aggregates as revealed by viscosity, dynamic light scattering (DLS), and small-angle neutron scattering (SANS) experiments. The critical micelle concentration was 6 mM (surface tension), with a hydrodynamic radius of 30 Å (light scattering) and 25.4 Å (SANS), area per molecule of about 57.6 Å, and aggregation number of 84.

Solubilization of drugs in ASC-8 micellar dispersions was reported by Palma et al. [16]. Solubilization of drugs was performed above the CMC and Krafft temperature determined by surface tension and conductivity measurements. The solubility of hydrophobic drugs, such as phenacetin, danthron, anthralin, and retinoic acid, was greatly enhanced by the solubilization. Furthermore, the antioxidant activity exhibited by the ascorbate rings that form the hydrophilic external shells in ASC-8 micellar dispersions can protect degradable materials that have been solubilized in the internal hydrophobic micellar core from radical-initiated oxidation.

### 1.4. Encapsulation of Ascorbyl Palmitate (ASC-P) in Carriers

ASC-P has been used as a model drug for nanosized lipid carriers. As an antioxidant, ASC-P has been also used in the cosmetics, food, and pharmaceutical industries. Teeranachaideekul et al. reported the physicochemical characterization and in vitro release studies of ASC-P-loaded nanostructured lipid carriers (NLC gels) [17, 18]. NLC gels were prepared by a high-pressure homogenization technique using oil Labrafil M1944 and solid lipids such as cetyl alcohol (CA), Imwitor 900 (GMS), and nonionic hydrophilic white beeswax (BW). Nanosized particles <250 nm with low polydispersity indices were prepared although microscopic observation indicated that the nanoparticles were nonspherical. The encapsulation efficiency of ASC-P was almost 100%, and its zeta potential was less than −30 mV. Differential scanning calorimetry (DSC) and powder X-ray diffraction (PXRD) measurements indicated that the lipid in each formulation was recrystallized in the solid state, possessing a less-ordered structure compared to that of the bulk material. The release study of ASC-P from each formulation using Franz diffusion cells revealed that the lipid matrix type affects both the rate and the release pattern. The release rate was in the order of ASC-P-loaded BW > ASC-P-loaded GMS > ASC-P-loaded CA. In viscoelastic analysis, all formulations showed that the storage modulus (G′) was higher than the loss modulus (G′′) and that the phase angle was <45°, indicating that they possess more elastic than viscous properties. Thus, NLC gel can be used as a colloidal carrier for topical application.

Wittayasuporn et al. reported encapsulation of ASC-P into methyl ether-terminated poly(ethylene oxide)-4-methoxycinnamolyphthaloylchitosan (PCPLC) nanoparticles [19]. PCPLC is a UV-screening amphiphilic chitosan derivative and is able to self-assemble into nanoparticles. Encapsulation of ASC-P into PCPLC resulted in 689 nm particles with encapsulation efficiency of 84% at a 56% drug-loading rate. The encapsulated ASC-P showed significantly improved stability when examined by the ^1^H NMR method in which both the tautomerized and the oxidized ASC-P could be monitored. ASC-P-encapsulated PCPLC nanoparticles demonstrated no short-term cytotoxicity against the human skin melanoma A-375 cell line and no short-term skin irritation on human volunteers. Aqueous suspension of PCPLC nanoparticles successfully inhibited the growth of *Escherichia coli* ATCC 25922 and *Staphylococcus aureus *ATCC 25923. Thus, ASC-P-encapsulated PCPLC nanoparticles with a photoprotective property appeared to be applicable to topically applied photolabile drugs and cosmetics.

Yoksan et al. reported the encapsulation of ASC-P in chitosan particles by oil-in-water (o/w) emulsion and ionic gelation processes using sodium triphosphate pentabasic (TPP) as a cross-linking agent [20]. ASC-P encapsulation was confirmed using conventional evaluation instruments: Fourier-transform infrared (FT-IR), ultraviolet-visible (UV-vis) spectrophotometer, thermal gravimetric analysis, and PXRD. The morphology of ASC-P-loaded chitosan particles was spherical with an average size of 60–100 nm as observed by scanning electron microscopy (SEM) and 30–60 nm by transmission electron microscopy (TEM). The loading capacity (weight of loaded ASC-P/weight of sample) and encapsulation efficiency (weight of loaded ASC-P/weight of initial ASC-P) of ASC-P in the nanoparticles were about 8–20% and 39–77%, respectively, when the initial ASC-P concentration was in the range of 25–150% (w/w) of chitosan. Release of ASC-P from the nanoparticles was explained by the loss of the cross-linked structure via electrostatic interaction between ammonium ions on chitosan chains and phosphoric groups of TPP molecules due to the deprotonation of chitosan in Tris buffer (pH ~ 8).

### 1.5. Stability of ASC-P in Carriers

ASC-P is a promising antioxidant candidate; however, its practical use is restricted because of its oxidation-induced poor solubility and instability. Kristl et al. reported that the stabilizing effect of carrier systems for ASC-P was investigated using microemulsions (ME), liposomes, and solid lipid nanoparticles (SLNs) [14]. ASC-P was resistant against oxidation in the order of nonhydrogenated soybean lecithin liposomes, SLN, w/o and o/w ME, and hydrogenated soybean lecithin liposomes. The location of the nitroxide group of ASC-P in a carrier system is crucial to its stability. Üner et al. compared the stability of ASC-P loaded in SLN, nanostructured lipid carriers (NLCs), and nanoemulsions (NEs) [15]. The highest level of degradation was observed with NE at all storage temperatures. These results indicated that the carrier structure is important to the maintenance of ASC-P stability. The degree of skin moisturizing and penetration of ASC-P entrapped in SLN, NLC, and NE incorporated into hydrogel was significantly higher compared to that of NE [21]. Enhanced stability of ASC-P encapsulated in poly(D,L-lactide) (PLA) and poly(D,L-lactide-co-glycolide) (PLGA) nanoparticles was reported by Tangsumranjit et al. [22]. In ASC-P-encapsulated PLA nanoparticles, ionic strength, and the degassing step affected ASC-P stability. Use of the PLA nanoparticle is a promising formulation for ASC-P stabilization.

### 1.6. ASC-P Nanosuspension

Teeranachaideekul et al. investigated the feasibility of applying nanosuspension technology by high-pressure homogenization (DissoCubes technology) to enhance the chemical stability of ASC-P, followed by lyophilization [23]. Sodium dodecyl sulfate (SDS) and Tween 80 were used as emulsifying agents to stabilize the developed ASC-P nanosuspensions. Tween 80 more effectively stabilized the nanoparticles than SDS. The percentage of ASC-P remaining in the nanosuspensions stabilized with Tween 80 was >90% after 3-month storage at 4°C, 25°C, and 40°C. The mean size of the ASC-P nanosuspensions prepared by redispersion of the lyophilized product was significantly higher compared with the system using 2–10% trehalose as a cryoprotectant. DissoCubes technology appeared to be effective in preparation of ASC-P nanoparticles using the optimum formulation.

### 1.7. Therapeutic Uses of Ascorbyl n-Alkyl Fatty Acid Derivative-Incorporated Nanocarriers

Representative applications of ASC-P for therapeutic uses include the skin permeation enhancer and synergistic cytotoxic action. Skin permeation enhancement of ASC-P by liposomal hydrogel (lipogel) formulation was reported by Lee et al. [24]. The ASC-P-incorporated liposome was formulated as lipogel by dispersion of the liposome into a poloxamer hydrogel matrix. The permeated amounts of ASC-P from the lipogels were higher than that of the control hydrogel containing Transcutol to solubilize ASC-P. Skin permeation of ASC-P improved when an electric current system that mimics an electric skin massager was used. In the cathodal delivery condition, the skin permeation characteristics of the negative lipogels were superior to those obtained with the neutral lipogels.

D'Souza et al. reported anticancer toxicity of ASC-P-incorporated liposomes and micelles in numerous cancer cell lines [25]. ASC-P-incorporated liposomes preferentially associated with various cancer cells compared to noncancer cells. In addition, ASC-P enhanced the cytotoxic action of paclitaxel when simultaneously incorporated into liposomes. The tumor-cell association and killing and the cytotoxic action of encapsulated paclitaxel in liposomes can potentiate the effect of ASC-P and paclitaxel. Cancer cell cytotoxicity and targeting was also observed both in vitro and in vivo using polyethylene glycol phosphatidylethanolamine micelles [26]. The mechanism of cell death was reported to be due to generation of reactive oxygen species.

Similar cytotoxic activity against tumor cells was reported using polymeric nanoparticles containing the antitumor compound transdehydrocrotonin (DHC) and L-ascorbic acid 6-stearate (ASC-S), which was taken up more easily by tumor cells than by normal ones [27]. ASC-S-DHC nanoparticles were prepared by the nanoprecipitation method, in which PLGA, DHC, and ASC-S dissolved in acetone were added to an aqueous solution containing Pluronic F68 and polyvinyl alcohol (PVA). ASC-S-DHC nanoparticles showed high drug loading efficiency (81–88%) and negative zeta potential (−29 to −32 mV) due to ASC-S. DHC-loaded nanoparticles demonstrated sustained release behavior. ASC-S-DHC nanoparticle suspension was a more effective antitumor agent than free DHC or DHC nanoparticles. Apoptosis induction of an ASC-S-DHC nanoparticle suspension was increased by the receptor-mediated pathway.

Gopinath et al. reported on the formation, characterization, and applications of ASC-P vesicles (Aspasomes) [28]. Submicron-sized Aspasomes were prepared using a film hydration method. A lipid film composed of various molar ratios of ascorbyl palmitate and cholesterol (27/63 to 72/18) and dicetyl phosphate at 10 mol% of total lipid was hydrated with phosphate buffered saline (PBS, pH 7.4). Aspasomes could encapsulate the hydrophilic drug zidovudine (AZT) when the film was hydrated with AZT-containing PBS. The suspension was then sonicated with an ultrasonicator for preparation of AZT-encapsulated aspasomes. The cholesterol content of the Aspasomes was not related with vesicle size, zeta potential, or percent of AZT entrapment. The release rate of AZT was changed by variation of the proportion of cholesterol, although there was no relation between release rate and cholesterol content. The antioxidant potency of ascorbyl moiety was retained even after the conversion of ascorbyl palmitate into an aspasome, and it rendered much higher antioxidant activity than ascorbic acid. The aspasome also showed enhanced skin permeation and retention properties of AZT, likely due to a role of ASC-P as a permeation enhancer.

### 1.8. Ascorbyl 2,6-Dipalmitate (ASC-DP)

**Figure 4:**
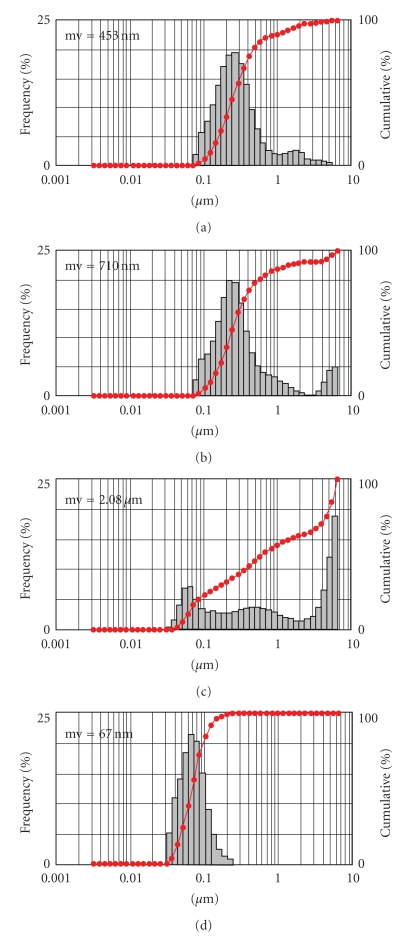
Particle size distribution patterns of ASC-DP/surfactant (1 : 1 molar ratio) suspensions. The surfactants included (a) SDS, (b) CTAB, (c) Brij78, and (d) DSPE-PEG.

ASC-DP has been used in the field of cosmetics [29, 30] and shows extremely low water solubility. In contrast with ASC-P, ASC-DP cannot form micelles or liposomal structures on its own; however, the ASC-DP-distearoylphosphatidylethanolamine-polyethylene glycol 2000 (DSPE-PEG) complex forms stable nanoparticles (Figure 4). Moribe et al. prepared drug-containing ASC-DP/DSPE-PEG nanoparticles and investigated their physical stability [31]. Many poorly water-soluble drugs were incorporated into the nanoparticles. Amphotericin B (AmB), a polyene macrolide antibiotic drug used to treat systemic invasive fungal infection, was selected as the model drug because it specifically interacts with DSPE-PEG [32]. This intermolecular interaction is believed to contribute to the effectiveness of AmB incorporation into the ASC-DP/DSPE-PEG nanoparticles. The stability, toxicity, and blood residence of the AmB/ASC-DP/DSPE-PEG nanoparticles was also investigated. The minimum lethal dose of Fungizone, a formulation of AmB solubilized with sodium deoxycholate, was 3.0 mg/kg, while that of AmB/ASC-DP/DSPE-PEG nanoparticles was 10.0 mg/kg in a formulation that was intravenously administered to mice. Intravenously administered AmB/ASCDP/DSPE-PEG nanoparticles were detected at higher concentrations than Fungizone in plasma. Thus, the ASC-DP/DSPE-PEG nanoparticle system appears to be a promising delivery system for hydrophobic drugs.

## 2. Conclusions

Formulation of hydrophilic and hydrophobic drugs using a nanosized carrier system is a promising way to achieve the desired therapeutic effect. Nanoparticle formation of ascorbic acid derivatives with or without drugs is practically applicable as transdermal and injectable formulations. The ascorbic acid derivatives shown in this paper can possibly be used as a model drug, a component of the carrier, or both. Ascorbic acid derivatives have been widely used as antioxidative drugs, the activities of which are similar to that of ascorbic acid. When combined with other excipients, such as oil with lipids and chitosan derivatives, several kinds of ASC-P-incorporated nanoparticles were formulated. Ascorbic acid and its derivatives are also used as cytotoxic drugs against cancer cell lines. Ascorbic acid derivatives with an alkyl chain are preferred because of the interaction with and insertion into the hydrophobic part of the membrane. This combined use with anticancer drugs incorporated in the carrier system apparently increased the efficacy. 

**Figure 5:**
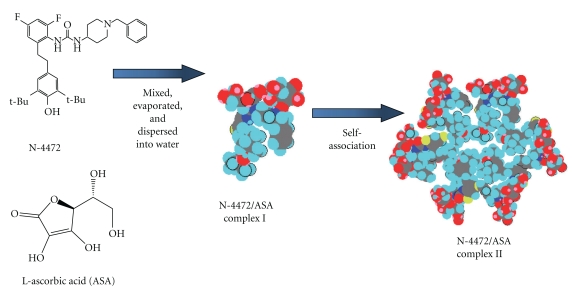
Schematic representation for proposed structure of N-4472/ASA surface active complex formation and the subsequent self-association used to form the stable nanosuspension.

A formulation design based on the chemical structure of the components is required not only to prepare stable drug nanoparticles but also to the broader application of ascorbic acid derivatives in therapeutic uses. The intermolecular interaction between each component and ascorbic acid derivatives contributes to effective drug solubilization and stabilization to enable nanoparticle formulation. For example, aspasomes were formulated as vesicles composed of various components that interacted with each other [28]. Furthermore, the complex formation between the hydrophobic drug N-4472 and ascorbic acid and the subsequent self-association contributed to form the colloidal particles in aqueous solution (Figure 5) [33]. A novel drug delivery carrier system using ascorbic acid derivatives is going to be developed using this chemical structure-based design.

Physicochemical characterization of the colloidal particles is important for understanding the drug absorption mechanism and therapeutic efficacy, although it is not easy. The molecular mobility measurement of a drug in aqueous media using NMR is useful to evaluate the molecular states of the drug in the formulation as well as in the dispersing media. This mechanistic study revealed the role of ascorbic acid derivatives in vitro and in vivo.

## Figures and Tables

**Table 1:** Physicochemical properties of ascorbic acid derivatives.

		Ref
ASC-G		
Solubility	714 g/L at 19 ± 1°C	*
Surface tension	71 mN/m (0.99 mg/mL)	*
Stability	Half-life at 50°C: >1 year at pH 4, 7, and 9	*
Toxicity	Acute oral toxicity (LD50): 2500 mg/kg (Rat)	*
	Acute dermal toxicity (LD50): >2000 mg/kg (Rat)	

ASC-8		
Critical micelle concentration	6 × 10^−3^ mol/L (pH2)	[1]
Hydrodynamic radius	25.4 Å	[1]
Head group area	57.6 Å^2^	[1]
Aggregation number	84	[1]

ASC-P		
Solubility	5.6 mg/mL at 25 ± 0.1°C	**
Toxicity	Acute oral toxicity (LD50): 25000 mg/kg (Mouse)	**
	Acute dermal toxicity (LD50): >3000 mg/kg (Guinea pig)	

ASC-S		
Solubility	Insoluble in water, soluble in ethanol	***

ASC-DP		
Solubility	Insoluble in water	****
Stability	Stable in aqueous media at pH 4, 7, and 10	****
	Stable in methanol	****
Safety	Oral administration: up to 1.25 mg/kg body weight daily	*****

*2-O-*α*-D-glucopyranosyl-L-ascorbic acid, Full Public Report National Industrial Chemicals Notification and Assessment Scheme, file no. STD/1056, 2003.

**http://www.sciencelab.com/xMSDS-Ascorbyl_palmitate-9922973.

***http://www.fao.org/ag/agn/jecfa-additives/details.html?id=43.

****Unpublished data.

*****http://www.hc-sc.gc.ca/.

**Table 2:** Application of ascorbic acid derivatives.

	Ref
ASC-G	
Quasidrug principal ingredient in skin care products	[3]
Food additive	[3]
Medical additive in commercial cosmetics	[3]
Skin antioxidant	[10]
Prevention of sinusoidal endothelial cell apotosis in preserved graft	[10]
High stability against thermal and oxidative degradation	[9]
Rapid conversion to ascorbic acid by *α*-glucosidase in the blood	[10]
Solubilization of clarithromycin (CAM)	[11]
Nanoparticle formation of CAM	[11]
Stabilization of CAM nanosuspension	[11]

ASC-8	
Solubilization of phenacetin, danthron, anthralin, and retinoic acid	[12]
Solubilization capacity of anthralin: ASC-8 < −10 < −12 < −14 < −16	[12]

ASC-P	
Cosmetic ingredients	[5]
Solubilization of drug	[5]
Decrease viscosity of gel formulation	[5]
Skin moisturizing and penetration effect of ASC-P entrapped in SLN, NLC, and NE incorporated into hydrogel	[21]
Antioxidant	[17–20, 23]
Stabilization of ASC-P by encapsulation in PLA nanoparticles	[22]
Skin permeation enhancer	[24]
Cytotoxicity against cancer cell	[25, 26]
ASC-P vesicles (Aspasomes)	[28]

ASC-S	
Cosmetic ingredients	*
Cytotoxicity against cancer cell	[27]

ASC-DP	
Cosmetic ingredients	*
Antioxidant (skin whitening action)	[29]
Extended the stability of adhesive transdermal pharmaceuticals	[29]
Nanoparticle formation with DSPE-PEG	[31]
Drug encapsulation in ASC-DP/DSPE-PEG	[31]

*Final report on the safety assessment of ascorbyl palmitate, ascorbyl dipalmitate, ascorbyl stearate, erythorbic acid, and sodium erythorbate.

Int. J. Toxicol.,18,1-26 (1999).
